# Partnership disengagement from primary community care networks (PCCNs): A qualitative study for a national demonstration project

**DOI:** 10.1186/1472-6963-10-87

**Published:** 2010-04-02

**Authors:** Chia-Yi Liau, Cheng-Chieh Lin, Yung-Kai Lin, Blossom Yen-Ju Lin

**Affiliations:** 1 Taichung Hospital, Department of Health, Executive Yuan, Taichung, Taiwan; 2 School and Graduate Institute of Health Services Administration, College of Public Health, China Medical University, Taichung, Taiwan; 3 Department of Family Medicine, China Medical University Hospital, Taichung, Taiwan; 4 College of Medicine, China Medical University, Taichung, Taiwan; 5 Institute of Health Care Administration, College of Health Science, Asia University, Taichung, Taiwan; 6 Division of Cardiovascular Surgery, Taichung Veterans General Hospital, Taichung, Taiwan

## Abstract

**Background:**

The Primary Community Care Network (PCCN) Demonstration Project, launched by the Bureau of National Health Insurance (BNHI) in 2003, is still in progress. Partnership structures in PCCNs represent both contractual clinic-to-clinic and clinic-to-hospital member relationships of organizational aspects. The partnership structures are the formal relationships between individuals and the total network. Their organizational design aims to ensure effective communication, coordination, and integration across the total network. Previous studies have focused largely on how contractual integration among the partnerships works and on its effects. Few studies, however, have tried to understand partnership disengagement in PCCNs. This study explores why some partnerships in PCCNs disengage.

**Methods:**

This study used a qualitative methodology with semi-structured questions for in-depth interviews. The semi-structured questions were pre-designed to explore the factors driving partnership disengagement. Thirty-seven clinic members who had withdrawn from their PCCNs were identified from the 2003-2005 Taiwan Primary Community Care Network Lists.

**Results:**

Organization/participant factors (extra working time spend and facility competency), network factors (partner collaboration), and community factors (health policy design incompatibility, patient-physician relationship, and effectiveness) are reasons for clinic physicians to withdraw or change their partnerships within the PCCNs.

**Conclusions:**

To strengthen partnership relationships, several suggestions are made, including to establish clinic and hospital member relationships, and to reduce administrative work. In addition, both educating the public about the concept of family doctors and ensuring well-organized national health policies could help health care providers improve the integration processes.

## Background

Regional or local health partnerships have been widely introduced around the world to improve health effectiveness from the perspectives of policy or provider strategy [1-6]. The presumed benefits of forming health networks are supporting expertise development, arranging referrals, coordinating programs, undertaking projects, sharing common interests, and providing mutual support for managing common conditions [7]. Many studies have explored how to run a health network more successfully. For example, it has been argued that sustainability is key to partnership success for community care network collaboration [8]. That includes sustaining the relationships, commitments, knowledge, capability, values, and trust, generated from partnerships, as well as sustaining funding, staff, programs, policy changes, and the partnership itself [9,10]. Human barriers have been identified as potential factors in network partnerships, among them user acceptance, limited support, technical skillfulness, awareness, and resistance to change [11]. Perceived competition, leadership struggles and confusion about roles have also been argued as barriers to service collaboration among medical practitioners [12]. Yet another study has highlighted patient centeredness, role delineation for partners, partner dynamics, and partner structure as critical to the success of family physicians' teams' partnering [13].

A systematic review of the literature on care coordination in Australia, US, UK, New Zealand, Canada, and the Netherlands has identified structural arrangements as supporting coordination [6]. The inter-organizational dimensions of structure, process, boundaries and network self-evaluation having also been emphasized as network objectives and evaluation criteria [3,14]. Humphreys et al. [15] have proposed these successful environmental enablers for the regional model of primary health care: supportive health policy, federal-state relations, and community readiness, as well as these service requirements: workforce, organization and supply, funding, governance, management and leadership, linkages, and infrastructure.

The Primary Community Care Network (PCCN) Demonstration Project, a nationwide healthcare financing program funded by the Bureau of National Health Insurance (BNHI) in March 2003, aimed to change the behavior of outpatients by giving them the freedom to select their medical providers. The PCCNs in Taiwan consist of groups of clinic physicians whose medical practices are classified as family care. The clinics most cooperate with at least one hospital for patients' secondary or tertiary care. The collaborative clinic-to-clinic and clinic-to-hospital member relationships within the PCCNs are the major structures. Each PCCN has a headquarters - usually in one of the clinic facilities - to coordinate and integrate the network. Each PCCN consists of five to ten clinics; half of them should offer these services: general medicine, internal medicine, surgery, obstetrics and gynecology, pediatric, or family medicine. Specialty clinics, usually handling outpatients with mild illnesses and less complicated symptomologies than hospital specialties treat, may also join the PCCN demonstration project. Such clinics include Otolaryngology, Ophthalmology, Rehabilitation Medicine, Dermatology, and Psychiatry. The PCCNs provide their outpatients (i.e. members' patients) with extra benefits: filing personal and family medical/health information for assisting health maintenance and offering suggestions; access 24 hours a day, 7 days a week via medical consultation telephone lines when their family physicians are off; free medical brochures and health or medical lectures; reminders of health examination appointments; health education for chronic disease management, and so on. The BNHI funded these extra involvements at a cost of 100,000USD (= NT $3,500,000) for the first year and negotiated for the continued years for each PCCN [3,16].

It has been more than five years since the Taiwan Health Authority launched the health care reforms undertaken by the PCCN demonstration project. The PCCNs' top-down partnerships, which are centrally steered and government-mandated arrangements, might differ from bottom-up, locally self-generated and voluntary partnerships. Several evaluation indicators have been assigned by the BNHI and academic researchers to evaluate this demonstration project; they include service quality [17], integration coordination among partnerships [3], and field evaluation of the clinics that use benchmarking among peers [16]. From data are obtained about partnership relationships, but are especially needed about failed relationships in clinic-to-clinic and clinic-to-hospital coordination. Therefore, using qualitative methodology, this study conducted in-depth interviews to understand the reasons for partner disengagement in the PCCNs, that is, the PCCNs' member clinics withdrawn or change their original networks. Using community, network, and organization/participants as the conceptual framework [18], this study yields findings that may help health policy makers and health care providers understand the difficulties that can hinder collaboration among hospitals and clinic physicians. The findings can also be used to help establish better relationships in physician-hospital integration.

## Methods

This is a qualitative study aiming to understand what factors drove the disengagements among/between clinic physicians and hospital members in PCCNs. The study was approved by the Taiwan National Science Council and the approval of Institutional Review of Board was obtained from China Medical University. The principal investigator signed the agreement to secure and obey all the requirements in academic ethics in study processing, study participants' confidentiality, and study findings and publication. In the non-experimental design using informants, the individual clinic physicians who have withdrawn their practices from the national demonstration project or changed their original network partners, informed consents were obtained by telephone in advance. All the respondents had agreed to our interviews, which we conducted one-on-one.

Qualitative methodology was used in this study. This is the first time the national health authority in Taiwan has launched a demonstration project to integrate contractual clinic-to-clinic and clinic-to-hospital member relationships. In the project's case reports, no experiences have been shared about the members' reasons for withdrawing or changing partnerships. Hence we have tried to collect as many thoughts as possible that might cast light on complex contents and relationships.

### Study subjects

At the end of 2005, the BNHI put into effect disciplinary regulations for those PCCN members who had not met such target indicators in health network management as not achieving the required referral rates, collecting enough patient members, and so on. Therefore we examined only the partnership changes in that occurred from March 2003 through September 2005, since the providers could "voluntarily" exit physician-hospital relationships during this period. As of September 2005, 53 clinic physicians had changed their status in their PCCNs - six clinic physicians changed their network partnerships, and 47 clinic physicians had withdrawn from their PCCNs. We verified these clinic physicians as our study population with the facility lists provided by the BNHI as well as our telephone checks.

To secure the privacy and rights of the 53 qualified study subjects, we first solicited their willingness to participate in telephone interviews. This led to the interviewing of 37 clinic physicians. Among those, four physicians were changing their partnership to other PCCNs, and 33 had withdrawn from their PCCNs and no longer participated in any partnership. The specialties of the 37 clinic physicians interviewed were: 11 in general medicine, six in Ophthalmology, seven in Obstetrics & Gynecology, two in Dermatology, four in Family Medicine, four in Internal Medicine, two in Surgery, and one in Otolaryngology. Nine physicians were with clinics in the northern area, sixteen in the central area, ten in the southern area, and two in the eastern area.

### Study questions

The study used in-depth interviews with semi-structured questions to help the study subjects recall and share their experiences with the research teams, and to aid in processing the interviews. Community, network, and organization/participants were used as the conceptual framework [18]. Community refers to the environments around the PCCNs, comprising patients, community, and political and health policy. Network refers to the functional coordination and relations of partnerships; and organization/participant refers to how the individual participants (network members) behave within PCCNs [3,19]. The questions included:

1. reasons to withdraw from PCCNs or change the original PCCNs;

2. challenges in collaboration among members in PCCNs from the perspectives of strategic planning, medical services planning and organization, information systems, financial involvement, integrated marketing, human resource management, policy regulation and constraints from organization, network, and community perspectives.

The predetermined questions were intended as a discussion guide [20] and also to probe for a better understanding of the study subjects' responses. Community, network, and organization/participants were the three categories for the transcripts of the informants' interviews.

### Data collection

The study conducted face-to-face or telephone interviews only after obtaining the informed consent of the clinic physicians. The processes of all interviews were taped, and the texts were transcribed word-for-word. To validate the accuracy of the interview transcripts, we double-checked with the interviewed clinic physicians and also asked some former partners, that is, their previous clinic or hospital members, to verify the information [21].

### Qualitative analysis

Grounded Theory was the basis for the methodology to explore the themes of partnership disengagement [22,23]. Qualitative inquiry was guided by the predetermined questions based on the previous literature, experiences, and interests, as a probe. They could induce some unstructured lines of response from participants to provide a deeper understanding. These in-depth interviews were the major source of the texts used in this study. All the interviews were audio-taped and transcribed. The texts were thematically coded, discussed, and categorized [22,23] by the authors, peers and participant validation. The frequency with which respondents endorsed various themes indicated the issues that emerged with regard to disengagement from partnership. The finals were presented in the following by the themes in the Results.

## Results

Following the initial readings of transcripts about withdrawing or changing partnerships within the PCCNs, the thematic categories of organization/participants, network, and community were derived. The results are as follows.

### Organization/participant factors: competency of clinics (physicians) in PCCNs

Organization/participant factors refer to how the clinic physicians' personal cognition, attitudes and behaviors influenced their decisions to leave or change their partnerships.

Time management was one of the first difficulties for clinic physicians. Most physicians who had withdrawn from PCCN partnerships mentioned the burden of the extra time required. They emphasized how the administrative and paper work threatened the time available for medical care in their working routines. They believed it might even threaten their health.

In our clinics, we have several medical service lines to provide for patients who pay out of pocket. We are too busy to take other administrative work. (Ophthalmology, central Taiwan)

Working time has filled up most of my time from eight in the morning to nine at night! (General practice, northern Taiwan)

I have no time to deal with administrative work, especially computer work. (Ophthalmology, eastern Taiwan)

Time pressures also resulted from the labor shortage in the small-scale clinics.

We are not like hospitals with sufficient labor to handle the administrative work. In the clinic business, it is difficult to manage the extra time and labor! My office hours start at seven in the morning and I have appointments until eight at night! (General medicine, northern Taiwan)

In addition to time spent on administrative work for the PCCN partnerships, the BNHI requires the participating clinic physicians to have family physician certificates, which means that time must be spent on continuing educations.

Training programs included in the demonstration project are useful for me. However, I have no time to participate in the education programs, because they were conflicting with my office hours. I have difficulties in handling the regulations proposed by the demonstration project. (Obstetrics &Gynecology, northern Taiwan)

The condensed training lectures and meetings make me grow in my medical knowledge. However, I had to sacrifice my leisure time. I just think I am always too tired and it might lead to a bad quality of patient care. I hate Wednesday! It is the day I feel exhausted! (General practice, southern Taiwan)

I have no time! There are too many time burdens in this demonstration projects.... united office hours, education programs... these extra time expenditures, and I have to sacrifice my time for taking care of my family. This is the reason I dropped out! (General practice, northern Taiwan) (Family medicine,  southern Taiwan) (Dermatology, central Taiwan)

Although the aim of training the member clinics' physicians to carry the abilities of family physicians is good, some physicians did not agree with it because they have been specialty-oriented in their careers for a long time.

It has been twenty to thirty years that I have focused only on my specialty (Obstetrics). It is hard to talk with patients with high blood pressure or something about pediatrics. ..... It takes away my confidence! This makes me want to quit this partnership. (Obstetrics &Gynecology, central Taiwan)

The demonstration project focused primarily on family medicine. My specialty (Ophthalmology) is more independent, and I just feel I cannot be helpful in this demonstration project. (Ophthalmology, central Taiwan)

Some broken partnerships resulted from the limited capabilities of the physician clinics, for system establishment, network collection, information infrastructure, data uploads to the BNHI, and computer operations.

### Network factors

Network factors (partnership relations) refer to how the clinic-clinic and clinic-hospital collaboration deterred the running of a health care network.

#### Clinic-to-clinic collaborative relationship deterred

Some physicians thought the collaboration within PCCNs was difficult from the very beginning. The diverse positions deterred the establishment of new relationships.

... *I am not familiar with the physicians around here. At the very beginning, it really bothered me. However, when I got into it, it was ok for me. (General medicine, southern Taiwan)*

... *different styles (culture) and objectives in management.... it is the reason that I withdrew from the partnership! (Internal medicine, eastern Taiwan)*

... *some of our members do not agree with the proposed ideas. (General medicine, northern Taiwan)*

... *some members asked new partners to come in... it broke our original proposed idea, and everything came again and again! (General medicine, northern Taiwan)*

... *different motivations and willingness among the partners make the collaboration more difficult. I feel sorry about this! (General medicine, northern Taiwan)*

Clinical service redundancy is another reason for the break-up of partnership. It impedes establishment of trust between partners.

We have had similar specialties for the past time within the network. It makes no sense to transfer the patients among member clinics within a network. (Family medicine, southern Taiwan)

#### Clinic-to-hospital collaborative relationship deterred

Some physicians argued that the hospital partners could dominate the collaboration in the demonstration project. Too much involvement by the member hospitals damaged work relationships.

... *our hospital (member hospital in a PCCN) is too strict to us.... they make demands to us (clinic physicians) about education hours, the number of referred patients..... they (member hospitals) are using us to expand their patient volume! (General medicine, southern Taiwan)*

The purpose of opening office hours in the partner hospital is for clinic physicians to take care of their patients previously referred to hospitals. However, given the hospital volume requirements from the BNHI, the lack of specified personnel to care for the referred patients and the extra time spent on office hours by the clinic physicians are pitfalls of the collaboration between clinic and hospital partners.

To open office hours in the member hospital, I have to sacrifice a lot of things, including my leisure time. I have to spend time learning to use the hospital computer systems. It is simply that I give up the time to treat my patients in my own clinic office. (Obstetrics &Gynecology, southern Taiwan)

It is not reasonable for me to schedule office hours in the hospital. I went there on Saturday afternoon. How could I find any hospital attending physicians to consult with at that time? (General medicine, northern Taiwan)

It is weird to have my office hour arranged at lunch time. Furthermore, it (the partner hospital) asked us to bring our patients there. I did not like that and neither did the patients. So, I quit! (Obstetrics &Gynecology, northern Taiwan)

... *I feel our member hospital did not care about our patients. My clinic is a little far away from our member hospital. I know that our member hospital did not have the full-time personnel to handle the transferred patients from the clinics in our health network..... Finally, we changed our hospital partner! (Family medicine, northern Taiwan)*

Several physicians mentioned that the partner hospitals are far away from the community they are familiar with. For example, at the request of the BNHI, some physicians referred their patients to member hospitals that were far away from their community. Yet, there are medical centers in their community! It makes patients confused. Moreover, some patients used to visiting certain hospitals and are unwilling to be assigned to the PCCN's hospital partner.

### Community factors: environmental enablers and disablers

Community factors for the regional model of primary health care could be environmental enablers and service requirements. Some clinic physicians argued about what a family physician is and what the differences are between Western countries and Taiwan. The unique characteristics of the Taiwan health care system include the high density of medical care facilities in the cities and counties. This weakens the obligation for Taiwanese citizens to follow the so-called primary, secondary and tertiary stages of medical care to use health services effectively. Ten physicians criticized the concept and design behind this PCCN demonstration project.

It should not be so complicated in medicine! I just want to serve my patients and that is why I am disappointed! Family physicians should be involved more in their patients' health. However, I do not think the BNHI is doing so! (Family medicine, Southern Taiwan) (General practice, southern Taiwan)

We (physicians) should do more about patient care, not the administrative work. It is not the job of physicians to recruit patients. The national authority should educate the Taiwan citizens about how to choose their physicians. The public health professionals could be good at this, and they should help to do this. (Ophthalmology, central Taiwan)

Furthermore, the freedom of Taiwanese citizens also deterred the implementation of clinic-hospital relationships. When the clinic physicians in a PCCN are assigned the role of "family physician," they start to recruit their patients to become PCCN patient members. The BNHI requires the participating physicians to recruit a certain volume of patients as members in the demonstration project. Some physicians thought it was difficult to persuade their patients to become members. Moreover, the number of member patients accumulated says nothing about the quality of care by the physicians.

I spent time persuading my patients. However, when I could not achieve the required member patient volume, I lost my authority! I just do not know what this number (recruited patients) stands for. (Ophthalmology, central Taiwan)

As requested by the BHNI, we have to recruit at least ten percent of our routine patients. So what? Can we only take care of that 10% of our routine patients, because they are our patient members? How about those 90% who are not recruited by us? (Family medicine, southern Taiwan)

Sometimes, it is not reasonable to ask for your patients to tell you about everything that has happened to them and their family. They just come for a simple cold. They (patients) were not willing to tell you something in privacy. The personal and family medical history information should only be collected for chronic patients, not for everyone. (Family medicine, southern Taiwan)

I cannot get used to "promoting" myself to patients to persuade them to become members. I just feel uncomfortable. (Orthopedics, southern Taiwan)

Citizens (patients) are the key.... Our national health authority has to educate the Taiwanese people about the advantages of family physicians and how good work can be done through physician-hospital coordination. When the Taiwanese people can recognize this, it is the only way to succeed, rather than just expecting what the health care providers should do. (General medicine, northern Taiwan)

Actually, patient-physician relationships take time to establish. Such relationships are not driven only by the PCCN demonstration project; they are long-term, and whether or not physicians participate in the demonstration project does not influence the patient-physician relationships. At times, the burden of paper work for physicians and member patients damaged the relationships.

It (patient volume) is not decreasing since I withdrew from the partnership. My patients are not running away because I am or am not a member of the demonstration project. It makes me realize that this country seems to be wasting the money! (General practice, southern Taiwan)

Patients are going with the physicians they trust! (Family medicine, southern Taiwan)

The required signatures for assuring member patient qualifications might destroy the relationship between me and my patients! (Family medicine, southern Taiwan)

Some physicians also mentioned that they were concerned about the effectiveness of PCCNs. For example, the participating physicians recruited their member patients and collected the members' data, including personal and family medical records, prevention services, disease management and so on. However, when these data were uploaded to the Bureau of National Health Insurance, no feedback was obtained.

What are the real purposes in this demonstration project? Who is responsible and accountable? Who (the patients) needs this demonstration project? We do not want do something that is not meaningful. (Ophthalmology, central Taiwan)

I cannot agree with releasing the data of patients. For example, the BNHI wants us to survey the member patients' personal and family medical histories for records. What about privacy? By the way, where is the patient information (data)? Now that I have withdrawn from the project, how do I deal with this patient information? (General practice, southern Taiwan)

I collected the prevention data for my member patients every day and uploaded to the national authority. However, what happens next? The national authority did not tell me what we can do next. The data collection seems to me like it is surveying and investigating "physicians". (Family medicine, southern Taiwan)

## Discussion

This study used semi-structured interview questions with qualitative methodology to understand the factors driving partnership disengagement. Organization/participant, network, and community factors of the conceptual framework [18] emerged as the reasons for clinic physicians to withdraw or change their partnerships in the PCCNs. The several reasons named include limited time for administration and paper work, the burden of continuing education, the partnership's limited capability for infrastructure, challenging partnership relationships, incompatibility of health policy design with PCCNs, strained physician-patient relationships, and systematic evaluation of partnership effectiveness. The results are summarized in Table 1. From clinic physicians in PCCNs, we learned that problems arose from the shifting requirements of their professional abilities and the heavy time management burden on their personal and family lives. There are serious challenges for clinic physicians in this PCCN demonstration project, especially given the circumstances of specialized medicine in the traditional health care system in Taiwan. Several respondents mentioned their difficulties on joining a primary care network because their careers had not included general medical experience. We may urge the health policy designers and decision makers to rethink the implications of participation by specialists from such fields as ophthalmology, dermatology, or otolaryngology in a PCCN.

**Table 1:** Summary of the reasons for withdrawing or changing partnerships within the PCCNs

Organization/participant factors
*1. working time required by the PCCNs*
*2. competency of clinics (physicians) in PCCNs:*
system establishment, network connection, information infrastructure, uploading data to the BNHI, computer operation, and shortage of labor to deal with the administrative work

**Network factors**
*1. clinic-to-clinic collaborative relationships deterred:*
diverse positions (parties), clinical service redundancy
*2. clinic-to-hospital collaborative relationships deterred:*
too many demands from hospital partners, few resource supports from hospital partners, large geographical distances between hospital and clinic partners

**Community factors**
*1. health policy design incompatibility* time needed to establish patient-physician relationships
*2. effectiveness of physician-hospital collaboration*

In order to avoid the pitfalls in traditional specialized medical education in Taiwan, the Department of Health has launched some strategies to strengthen physician education. One is called the "Postgraduate Primary Care Training Program" [24]. This program requires each physician in his/her first year of residency to fulfill one month of general medical training in primary care medicine, primary care surgery and community medicine, along with 36 hours of basic courses. The program was introduced in Taiwan to strengthen the general medical training of medical students after graduation. We believe this program will add to the knowledge and skills provided by medical education training when physicians start their practice.

More focus is needed on socialization in the atmosphere of PCCN partnerships, including the partners' cultures and behavior. Murray et al. [25] examined the primary care reform in Canada and found that although the health care providers reported themselves ready to make necessary changes and willing to move to interdisciplinary team practices, challenges still impeded that movement. Previous positive experience with partners is a key to motivating clinicians to join partnerships. Important facilitating factors are effective leadership, the aim of the project, and sharing vision and goals [26]. Drawing on previous empirical and managerial studies, Friedman and Goes [19] summarized several key issues: misalignment of culture and incentives, difficulties in building trust among network stakeholders, problematic leadership, uncertain visions of the desired outcomes and lack of commitment and understanding. These authors' model could serve as a mirror for the PCCN partnerships in Taiwan.

In terms of the clinical service designs in PCCNs, one might argue that the national authority should re-examine the locations of the PCCN (clinic and hospital) members. The aim should be to avoid dysfunctional competition among the clinic members due to service redundancy, as well as patient referrals to hospitals far from their community [27].

For patients, what we learned in this study is that time has to be spent to educate people about the advantages of family physicians and the disadvantages of shopping for hospitals. In Taiwan, people prefer to seek medical care at large-scale hospitals even when they get just a simple cold, because of a lack of confidence in clinic practices. We urge the national authority to educate people about the value of family physicians. It is also necessary to assist clinic physicians in managing the transitions of health care reform. Special attention should be paid to those clinic offices that might have a difficult time handling additional administrative or paper work because of their limited staff.

Another issue to consider is how the member patients' personal health records are used in PCCNs. As we mentioned earlier, the clinic physicians are required recruit member patients and obtain their consent for collecting relevant health information about them and their families. Some physicians have shown concern about privacy and security in the access to these patients' health information even after their physicians have withdrawn from network partnerships. Another concern is that member patients may inadvertently disclose their health information without understanding what signing the consent forms really means [28]. Some physicians also pointed out that patients with chronic diseases and those in racial and ethnic minorities have more basis for their concerns about the privacy of their health records [29]. From a public policy perspective, lack of trust about the privacy of patient health information is not only about the ownership of the data, but also about how the health authority might develop and promote use of the personal health records. To bridge the potential gap between the present information technology infrastructures and future national regulations, these concerns must be addressed.

This study interviewed participants who had left the network partnerships, but not those who had stayed in network relationships. Future studies could focus on the participants who stay in the network partnerships. Combining the opinions of partners who remain with this study's findings could enable confident conclusions. Moreover, since open-expression interviews with the providers about their experiences focus only on their personal and perhaps fragmentary comments, the whole picture could be enhanced by also considering the experts' assessment and understanding of the advantages and disadvantages of the demonstration PCCN project.

We were dealing with break-ups among the partners at an early stage. Mature partnerships might face further changes and different challenges, since they would engage more and broader staff and responsive mechanisms to make the partnership work [30]. It would be worth examining the partnership relationship in the long run to understand the effectiveness of primary care reform.

## Conclusions

Partner disengagement from PCCN demonstration projects could be attributed to an organization's/participant's involvements, capabilities, and administrative burdens. Furthermore, in the long run, the establishment of trust and mutual understanding and coordination may be essential for primary care networks that use partnerships both between clinics and clinics, and between clinics and hospitals. The study also sought to understand how the incompatibility of health policy design with this national PCCN demonstration project might influence the effectiveness of health care networks. The study findings can strengthen the understanding of organizations, health professionals, and health policy makers, to cast light on network situations and strengthen partnership relationships.

## List of abbreviations used

PCCN: Primary Community Care Network; BNHI: Bureau of National Health Insurance.

## Competing interests

The authors declare that they have no competing interests.

## Authors' contributions

BYJL designed and conducted this study. CYL interviewed all subjects and made the detailed records and transcriptions. CCL and YKL were involved in the study processes and discussions (data interpretation), and offered practical thoughts and networking for this study. All the authors reviewed, edited, and approved the final manuscript.

## Pre-publication history

The pre-publication history for this paper can be accessed here:

http://www.biomedcentral.com/1472-6963/10/87/prepub
